# miR-454 suppresses the proliferation and invasion of ovarian cancer by targeting E2F6

**DOI:** 10.1186/s12935-020-01300-0

**Published:** 2020-06-12

**Authors:** Yunhe An, Jun Zhang, Xiaoyan Cheng, Baoming Li, Yanjie Tian, Xiaoli Zhang, Fangqi Zhao

**Affiliations:** 1 Department of Biotechnology, Beijing Center for Physical and Chemical Analysis, No. 27 Xisanhuan North Road, Beijing, 100089 China; 2 Department of Obstetrics and Gynecology, Beijing Anzhen Hospital, Capital Medical University, Beijing, 100029 China

**Keywords:** Ovarian cancer, miR-454, E2F6, Growth, Metastasis

## Abstract

**Background:**

The aberrant expression of microRNA-454 (miR-454) has been confirmed to be involved in the development of cancers. However, the functional role of miR-454 in the progression of ovarian cancer remains unclear.

**Methods:**

The expression of miR-454 in ovarian cancer cells and serum of ovarian cancer patients was detected by RT-PCR. CCK8, colony formation, transwell, and flow cytometry assays were conducted to assess the effects of miR-454 on ovarian cancer cell proliferation, migration, invasion, and apoptosis, respectively. Dual-luciferase reporter assay was used to confirm the targeting relationship between miR-454 and E2F6. The expression pattern of E2F6 in ovarian cancer tissues was detected using immunohistochemistry (IHC) assay. The relative expression of related proteins was examined using western blot analysis.

**Results:**

miR-454 was markedly down-regulated by hypoxia in ovarian cancer cells. Compared with normal samples, the expression of miR-454 was up-regulated in the serum of ovarian cancer patients, and correlated with the clinicopathological stages of ovarian cancer. Next, we found that miR-454 overexpression inhibited the proliferation, migration and invasion of OVCAR3 and SKOV3 cells, as well as promoted apoptosis. In addition, the Akt/mTOR and Wnt/β-catenin signaling pathway were inhibited by miR-454 in ovarian cancer cells. Mechanically, bioinformatic analysis and dual-luciferase reporter assay confirmed that E2F6 was a direct target of miR-454 and negatively regulated by miR-454 in ovarian cancer cells. Moreover, IHC analysis showed that E2F6 was highly expressed in ovarian cancer tissues. Finally, we found that the increasing cell proliferation and migration triggered by E2F6 overexpression were abolished by miR-454 overexpression.

**Conclusion:**

Taken together, these results highlight the role of miR-454 as a tumor suppressor in ovarian cancer cells by targeting E2F6, indicating that miR-454 may be a potential diagnostic biomarker and therapeutic target for ovarian cancer.

## Background

Ovarian cancer has the highest mortality rate in gynecological malignancies, with approximately 140,000 deaths worldwide each year [1, 2]. There are three main types of ovarian cancer: epithelial, germ cell, and sex-cord-stromal, with more than 90% of ovarian cancer have epithelial histological features [3]. These subtypes are distinct in many aspects, including etiology, morphology, molecular biology and prognosis, but are all treated as a single entity [4]. Cytoreductive surgery and platinum/paclitaxel combination chemotherapy are the standard treatments for ovarian cancer [4]. However, most patients relapse and the 5-year survival rate for patients with ovarian cancer is still below 50% [5, 6]. Concealment of symptoms in early stages, chemotherapy resistance, and lack of effective early detection are the main factors that cause poor prognosis in patients with ovarian cancer [7]. Therefore, it is urgent to develop novel diagnostic biomarker and therapeutic target for ovarian cancer.

Increasing number of studies reveal that microRNAs (miRNAs) are closely involved in tumorigenesis and tumor progression [8–10]. miRNAs can negatively regulate expression of target gene by binding to the 3′-UTR of target gene to inhibit mRNA translation or promote mRNA degradation [11, 12]. A number of miRNAs have been proved to be dysregulated in ovarian cancer, and act as either tumor suppressor or promoter in the growth and metastasis of ovarian cancer [13–15]. More importantly, the miRNAs in serum are also closely related to malignant tumors, and are considered to be new diagnostic biomarkers due to their availability, high stability, and disease specificity [16]. miR-454 has been reported to be implicated in the progression of many types of cancer, playing dual roles in different tumors. Studies show that miR-454 functions as an oncogene in colorectal cancer [17], hepatocellular carcinoma [18] and non-small cell lung cancer [19], but servers as a tumor suppressor in osteosarcoma [20] and glioblastoma [21]. However, the function and mechanism of miR-454 in ovarian cancer remain largely unclear.

The results of the current study showed that miR-454 was up-regulated in serum of patients with ovarian cancer that the role of miR-454 in the growth and metastasis of ovarian cancer cells in vitro was analyzed. Mechanically, E2F6 was identified as a direct target of miR-454, which was up-regulated in ovarian cancer tissues and involved in the tumor suppressive role of miR-454. This study advances the understanding of the mechanism of ovarian cancer occurrence and development, and suggest that miR-454 may be a novel diagnostic biomarker for ovarian cancer, as well as a therapeutic target.

## Materials and methods

### Cell lines and cell culture

OVCAR3 and SKOV3 cells were obtained from Cell Bank of Chinese Academy of Sciences (Shanghai, China) and maintained in RPMI-1640 medium (HyClone, USA) supplemented with 10% FBS at 37 °C with 5% CO_2_. Cells were transfected with pCMV-MIR-miR-454 (5 μg; Ribobio, Guangzhou, China) using Lipofectamine 2000 (Invitrogen, CA, USA) according to the instructions, pCMV-MIR vector (5 μg; Ribobio) was used as negative control (NC). The E2F6 cDNA sequences were cloned into pcDNA3.1 vector and the pcDNA3.1-E2F6 (5 μg; Ribobio) was transfected into cells using Lipofectamine 2000.

### Clinical samples

Seventy-five cases of ovarian cancer tissues and 15 cases of tumor-adjacent tissues were obtained from Beijing Anzhen Hospital, Capital Medical University. All patients gave informed consent in written, and this study was approved by the ethics committee of Beijing Anzhen Hospital, Capital Medical University. Venous blood was collected from 13 patients with ovarian cancer and 6 normal female subjects from Beijing Anzhen Hospital, Capital Medical University. The patient samples and normal subjects age was matched. All blood samples were taken before patient treatment.

### Quantitative real-time polymerase chain reaction (RT-PCR) analysis

Total RNA extracted from OVCAR3 and SKOV3 cells or serum samples using TRIzol Reagent. The SYBR PrimeScript miRNA RT PCR kit (Takara, Shiga, Japan) was performed for RT-PCR reaction to examine the expression of miR-454. For detection of E2F6 mRNA expression, a HiFiScript cDNA Synthesis Kit (CWBIO, Beijing, China) was used to reverse transcribe RNA to cDNA, and a SYBR Premix Ex Taq II kit (Takara) was performed for RT-PCR reaction. Reaction conditions were as follows: 95 °C for 10 min, followed by 40 cycles of 95 °C for 5 s and 60 °C for 30 s. U6 or GAPDH was used as reference genes. Primers of miR-454 and E2F6 were obtained from Ribobio. The relative expression of miR-454 or E2F6 mRNA was calculated using the ^2−ΔΔCT^ method and normalized to NC group.

### Cell counting kit-8 (CCK8) assay

Cells were seeded in a 96-well plate at a density of 1000 cells/well and cultured at 37 °C with 5% CO_2_ for 0, 24, 48, and 72 h, respectively. Then, 10 μL of CCK8 reagent (Beijing Solarbio Science & Technology, Beijing, China) was added into each well. Following incubation at 37 °C for 1.5 h, the absorbance was detected at 450 nm.

### Colony formation assay

Cells were cultured in 60-mm dishes (500 cells/dish) and incubated at 37 °C with 5% CO_2_ for 1–2 weeks. After that, the colonies were fixed with 4% paraformaldehyde for 30 min at room temperature and dyed with 0.1% crystal violet for another 30 min at room temperature. Finally, the number of cell colonies was counted.

### Cell migration and invasion assays

Transwell chambers (Millipore, MA, USA) were used to measure cell migration and invasion. About 1 × 10^5^ cells were seeded in the upper chamber and the lower chamber was filled with RPMI-1640 medium containing 20% FBS. Following incubation at 37 °C of 12 h, the migrated or invaded cells were fixed with 4% paraformaldehyde for 30 min at room temperature, and stained with 0.1% crystal violet for 20 min at room temperature. In invasion experiment, the upper chamber was coated with Matrigel (BD Bioscience, CA, USA) before seeding cells.

### Cell apoptosis assay

Cells transfected with plasmids of 24 h were cultured in serum-free medium at 37 °C for 24 h, trypsinized by trypsin (0.25%) without EDTA and centrifuged at 1000 rpm for 5 min. After washing with PBS twice, cells were re-suspended in loading buffer to prepare 1 × 10^6^ cells/mL suspension. 100 μL cell suspension were incubated with 5 μL of Annexin V/FITC (BioVision, USA) for 5 min in the dark at room temperature, then stained with 10 μL propidium iodide (PI). The apoptotic rate was examined by a flow cytometer (BD FACSC anto II, BD Biosciences, USA) and analyzed using FlowJo v7.6.5 software (Tree Star, Inc., Ashland, OR, USA).

### Western blot assay

Total proteins were extracted from transfected cells by RIPA lysis, and quantitated by a BCA kit (CWBIO). Proteins (20 μg) were subjected to 10% SDS-PAGE and transferred onto the PVDF membranes (Bio-Rad, Hercules, CA, USA). After blocked with 5% non-fat milk solution for 1 h, the membranes were probed with primary antibodies overnight at 4 °C. After washing with TBST, the blots were incubated with horseradish peroxidase (HRP)-conjugated secondary antibodies (1:5000 dilution, SA00001-1, SA00001-2, Proteintech Group, USA) for 1 h at room temperature, and developed using the Enhanced Chemiluminescence kit (CWBIO). The band intensity was quantified using Image J software (NIH, Bethesda, USA). The primary antibodies used in this study were anti-Bcl-2 (1:1000 dilution, 12789-1-AP, Proteintech Group), anti-Bax (1:1000 dilution, 50599-2-Ig, Proteintech Group), anti-cleaved Caspase 9 (1:1000 dilution, ab2324, Abcam, USA), anti-cleaved Caspase 3 (1:1000 dilution, ab2302, Abcam), anti-Akt (1:1000 dilution, 10176-2-AP, Proteintech Group), anti-Akt-Phospho-S473 (1:1000 dilution, 66444-1-Ig, Proteintech Group), anti-mTOR (1:1000 dilution, 20657-1-AP, Proteintech Group), anti-mTOR (phosphor S2448) (1:1000 dilution, ab109268, Abcam), anti-Cyclin D1 (1:1000 dilution, 26939-1-AP, Proteintech Group), anti-p70s6k (1:1000 dilution, 14485-1-AP, Proteintech Group), anti-Phospho-p70s6k (1:1000 dilution, 9204, Cell Signaling Technology, USA), anti-Wnt3 (1:1000 dilution, 67452-1-Ig, Proteintech Group), anti-β-catenin (1:1000 dilution, 17565-1-AP, Proteintech Group), anti-E-cadherin (1:1000 dilution, 20874-1-AP, Proteintech Group), and anti-GAPDH (1:1000 dilution, 10494-1-AP, Proteintech Group).

### Dual-luciferase reporter assay

TargetScan (http://www.targetscan.org/vert_72/) [22] was used to explore the bindingship between miR-454 and E2F6. The wild-type of E2F6 3′-UTR (E2F6-wt) containing the predicated miR-454-bingding site or mutant type of E2F6 3′-UTR (E2F6-mut) was cloned into pmir-GLO reporter vector (Promega, WI, USA). 293T cells were co-transfected with luciferase reporter plasmids (wt or mut; 1 ng/μL) and 5 μg either pCMV-MIR-miR-454 or pCMV-MIR using Lipofectamine 2000. Following transfection of 48 h, cells were collected and lysed, the luciferase activity of each group was measured by the dual-luciferase assay system kit (Promega).

### Immunohistochemistry (IHC) analysis

IHC was performed to evaluate the expression of E2F6 protein (1:50; Abcam) as described previously [23]. E2F6 expression levels were scored by staining intensity and percentage of positive stained cells [24]. The staining intensity was graded as follows: 0, no staining; 1, pale yellow staining; 2, buffy staining; 3, intense brown staining. The percentage of positive stained cells was scored as follows: 0 = 0–10%, 1 = 11–25%, 2 = 26–50%, 3 = 51–75%, 4 => 75%. Samples were scored by multiplying synchronically the two sections, and score > 6 as E2F6 high expression level. Ovary tissue was used as positive control, and PBS was used as the negative control instead of primary antibody.

### Statistical analysis

Data was presented as Mean ± SD and statistical analyses were conducted using GraphPad Prism 7.0 software (GraphPad, CA, USA). The Chi-square test was performed for continuous or discrete data analysis, and the Student’s t test or one-way ANOVA analysis was used for comparisons between groups. Differences were considered significant when P value was less than 0.05.

## Results

### miR-454 is up-regulated in ovarian cancer and suppresses the proliferation, migration and invasion of ovarian cancer cells

**Fig. 1:**
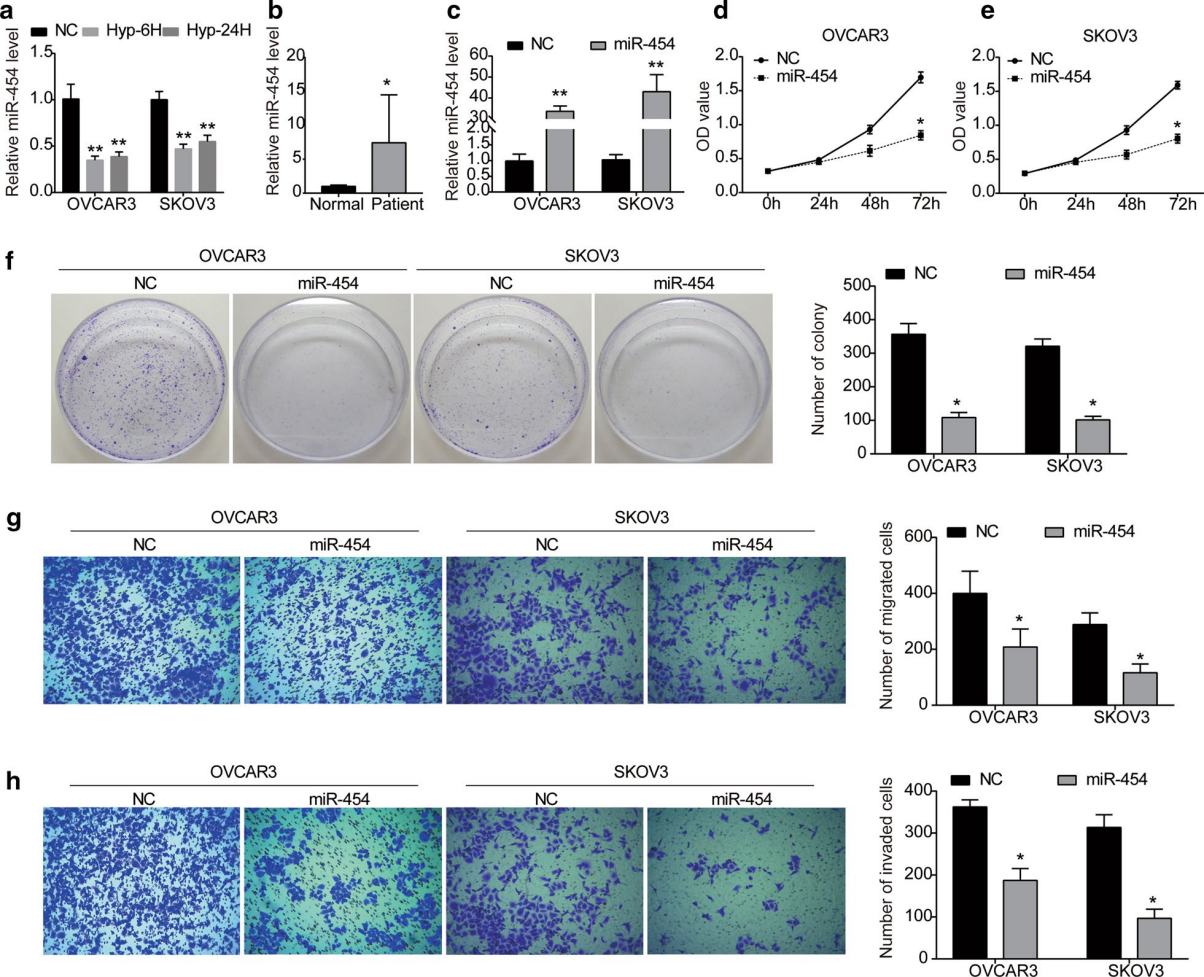
miR-454 overexpression inhibits the proliferation, migration and invasion of ovarian cancer cells. **a** Expression of miR-454 was examined in OVCAR3 and SKOV3 cells under hypoxic culture conditions (1% O_2_, 5% CO_2_, 94% N_2_) for 6 or 24 h. **b** Expression of miR-454 in the serum of ovarian cancer patients (n = 13) and normal samples (n = 6) was measured by RT-PCR. **c** Expression of miR-454 in OVCAR3 and SKOV3 cells transfected with either pCMV-MIR-miR-454 or pCMV-MIR plasmid was detected byRT-PCR. **d**, **e** CCK8 assay was used to examine the proliferation of OVCAR3 (**d**) and SKOV3 (**e**) cells after transfected with either pCMV-MIR-miR-454 or pCMV-MIR plasmid. **f** Colony formation assay of OVCAR3 and SKOV3 cells with stable miR-454 overexpression. **g**, **h** Transwell assay was performed to assess the migration (**g**) and invasion (**h**) abilities of OVCAR3 and SKOV3 cells with stable miR-454 overexpression. NC, cells transfected with pCMV-MIR plasmid; miR-454, cells transfected with pCMV-MIR-miR-454 plasmid. *P < 0.05, **P < 0.01 vs. NC group

To investigate whether miR-454 is correlated to the progression of ovarian cancer, we first examined the expression of miR-454 in ovarian cancer. We found that miR-454 expression was obviously down-regulated by hypoxia (after 6 or 24 h in 1% O_2_) in OVCAR3 and SKOV3 cells compared with corresponding normoxic condition (Fig. 1a). These data indicated the specificity of miR-454 in hypoxic induction in ovarian cancer. Next, we examined the expression of miR-454 in the serum of ovarian cancer patients (n = 13) and normal human serum (n = 6). The expression of miR-454 was found to be significantly elevated in the serum of ovarian cancer patients compared to normal samples (Fig. 1b). Additionally, our data demonstrated that the expression of miR-454 was correlated with the clinicopathological stages of ovarian cancer (*P *= 0.016), the expression of miR-454 in the serum of patients in stage I–II was significantly higher than that in stage III–IV (Table 1). But the expression of miR-454 was not found to be correlated with age and lymph node metastasis of ovarian cancer patients (Table 1). Therefore, miR-454 may be involved in the progression of ovarian cancer.

**Table 1:** The correlation between the expression of serum miR-454 and the clinicopathological characteristics of ovarian cancer patients

Characteristics	n	miR-454 expression	*P*-value
Age			
< 59	7	6.41 ± 5.33	0.69
≥ 59	6	8.37 ± 11.39	
Clinical stage			
I–II	7	12.13 ± 8.92	0.016*
III–IV	6	1.70 ± 0.77	
Lymph node metastasis			
Yes	7	5.69 ± 10.59	0.47
No	6	9.21 ± 4.88	

*P < 0.05

To elucidate the functional role of miR-454 in ovarian cancer, pCMV-MIR-miR-454 was transfected into ovarian cancer cell lines OVCAR3 and SKOV3, pCMV-MIR plasmid was used as negative control (NC). RT-PCR analysis showed that miR-454 expression was markedly up-regulated in OVCAR3 and SKOV3 cells transfected with pCMV-MIR-miR-454 (Fig. 1c). As shown in Fig. 1d, e, miR-454 overexpression significantly reduced the proliferation of OVCAR3 and SKOV3 cells compared with corresponding control group. Consistently, OVCAR3 cells transfected with pCMV-MIR-miR-454 formed fewer colonies than control group (Fig. 1f). Similar results were also observed in SKOV3 cells, up-regulation of miR-454 inhibited colony formation ability of SKOV3 cells (Fig. 1f). Moreover, transwell assay showed that up-regulation of miR-454 dramatically inhibited the migration and invasion abilities of both OVCAR3 and SKOV3 cells with a contrast to that of the NC group (Fig. 1g, h). These results suggest that miR-454 suppresses the growth and metastasis of ovarian cancer cells, exerting a tumor suppressor effect.

### miR-454 promotes cell apoptosis and inhibits the Akt/mTOR and Wnt/β-catenin signaling pathways in ovarian cancer cells

**Fig. 2:**
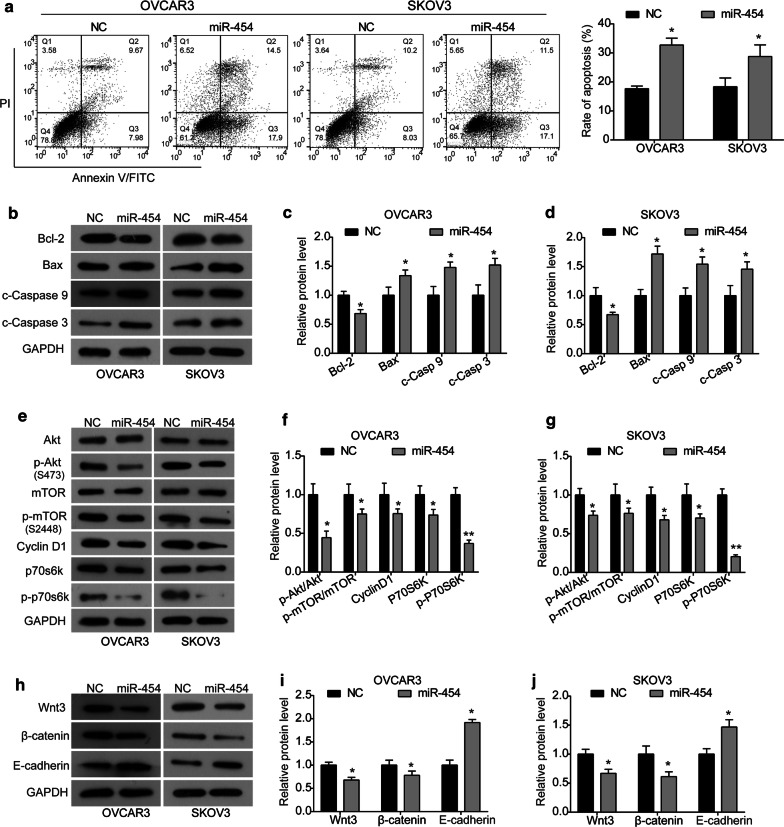
miR-454 overexpression promotes apoptosis of ovarian cancer cells and inhibits the Akt/mTOR and Wnt/β-catenin signaling pathways. **a** Apoptosis rate of OVCAR3 and SKOV3 cells transfected with either pCMV-MIR-miR-454 or pCMV-MIR plasmid was measured by flow cytometry assay. **b** Following transfection with either pCMV-MIR-miR-454 or pCMV-MIR plasmid for 48 h, the expression of apoptosis-related proteins (Bcl-2, Bax, cleaved Caspase 9, and cleaved Caspase 3) in OVCAR3 and SKOV3 cells was detected using western blot. **c**, **d** Quantitative analysis of Western blot results in **b**. **e** Expression of the Akt/mTOR signaling pathway related proteins in OVCAR3 and SKOV3 cells was detected using western blot. **f**, **g** Quantitative analysis of Western blot results in **e**. **h** Expression of the Wnt/β-catenin signaling pathway related proteins in OVCAR3 and SKOV3 cells was detected using western blot. **i**, **j** Quantitative analysis of Western blot results in **h**. *P < 0.05, **P < 0.01 vs. NC group

As indicated by flow cytometry assay, miR-454 overexpression significantly enhanced the apoptosis rate of both OVCAR3 and SKOV3 cells with a contrast to NC group (Fig. 2a). Then, the expression of apoptosis-related proteins was detected by western blot analysis to investigate the underlying mechanism of induced apoptosis by miR-454. As shown in Fig. 2b–d, the expression of anti-apoptotic protein Bcl-2 was significantly down-regulated by miR-454 in both OVCAR3 and SKOV3 cells, while the expression levels of pro-oncogenic proteins Bax, cleaved Caspase 9 and cleaved Caspase 3 were up-regulated by miR-454. The above data indicate that miR-454 may induce apoptosis in ovarian cancer cells by regulating the Bcl-2/Bax axis and Caspase cascade.

It is well known that the Akt/mTOR and Wnt/β-catenin signaling pathways play critical roles in tumorigenesis and development. To further investigate the role of miR-454 in ovarian cancer, we examined the effect of miR-454 on activation of the Akt/mTOR and Wnt/β-catenin signaling pathways in ovarian cancer cells. As indicated by western blot analysis, the phosphorylation levels of Akt (p-Akt) and mTOR (p-mTOR) were reduced in miR-454 group compared with NC group (Fig. 2e–g). Additionally, the expression of downstream proteins Cyclin D1 and p70s6k was inhibited correspondingly in miR-454 group, as well as the level of phosphorylation form of p70s6k (p-p70s6k) (Fig. 2e–g). Similarly, the expression levels of Wnt3 and β-catenin were also down-regulated by miR-454 overexpression in OVCAR3 and SKOV3 cells, the expression of downstream protein E-cadherin was up-regulated correspondingly (Fig. 2h–j).

### E2F6 is a direct target of miR-454 in ovarian cancer cells

**Fig. 3:**
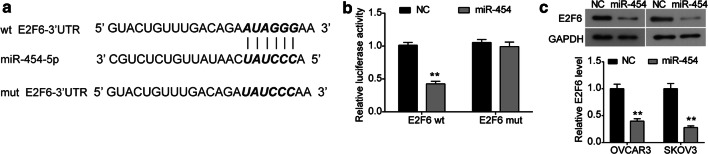
E2F6 is a direct target of miR-454 in ovarian cancer cells. **a** The predicted binding site for miR-454 in the 3′-UTR of E2F6 mRNA was revealed by TargetScan. **b** Relative luciferase activity in cells co-transfected with pCMV-MIR-miR-454 or pCMV-MIR and pmir-GLO-E2F6 wild type (wt) or pmir-GLO-E2F6 mutant (mut) was measured by dual-luciferase reporter assay. **c** The protein expression of E2F6 was checked in OVCAR3 and SKOV3 cells transfected with either pCMV-MIR-miR-454 or pCMV-MIR plasmid by western blot. **P < 0.01 vs. NC group

In order to elucidate the molecular mechanism of miR-454, we identified the potential target of miR-454. Bioinformatics analysis (TargetScan) suggested E2F6 mRNA had a possible binding site for miR-454 in its 3′-UTR (Fig. 3a). To confirm that, the wild-type (wt) or mutant-type (mut) E2F6 3′-UTR was cloned into a luciferase reporter vector, which was co-transfected with pCMV-MIR-miR-454 into 293T cells. As shown in Fig. 3b, the relative luciferase activity of E2F6-wt was markedly decreased in the presence of miR-454, while the luciferase activity of E2F6-mut was not impaired by miR-454. Moreover, the expression of E2F6 protein was significantly inhibited in OVCAR3 and SKOV3 cells transfected with pCMV-MIR-miR-454 (Fig. 3c). Therefore, E2F6 was a direct target of miR-454 in ovarian cancer cells.

### E2F6 is up-regulated in ovarian cancer and involved in the effects of miR-454 on ovarian cancer cells

**Fig. 4:**
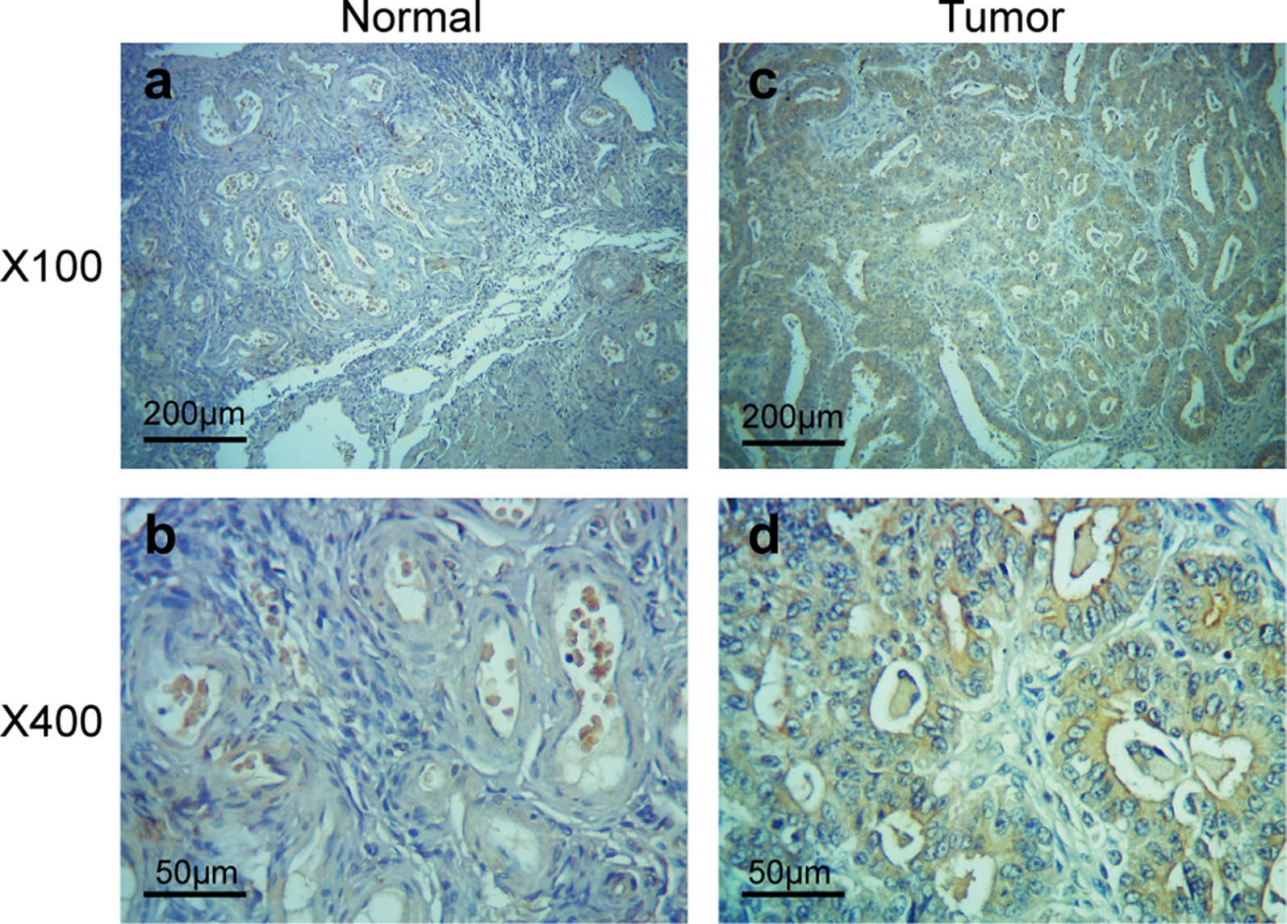
E2F6 is highly expressed in ovarian cancer tissues. IHC analysis was performed to detect the expression of E2F6 protein in ovarian cancer tissues (Tumor) and tumor-adjacent tissues (Normal). **a** Tumor-adjacent tissue (original magnification ×100). **b** Tumor-adjacent tissue (original magnification ×400). **c** Ovarian cancer tissue (original magnification ×100). **d** Ovarian cancer tissue (original magnification ×400)

The IHC analysis was performed to determine the expression of E2F6 protein in 75 cases of ovarian cancer tissues and 15 cases of tumor-adjacent tissues. As shown in Fig. 4a–d, the positive signal of E2F6 was found to mainly located in nucleoplasm. The rate of E2F6-high expression in ovarian cancer tissues was 57.3% (43/75), which was 20% (3/15) in tumor-adjacent tissues (*P *< 0.05). Additionally, we observed that E2F6 protein was highly expressed in ovarian cancer tissues compared with tumor-adjacent tissues (Fig. 4a–d).

**Fig. 5:**
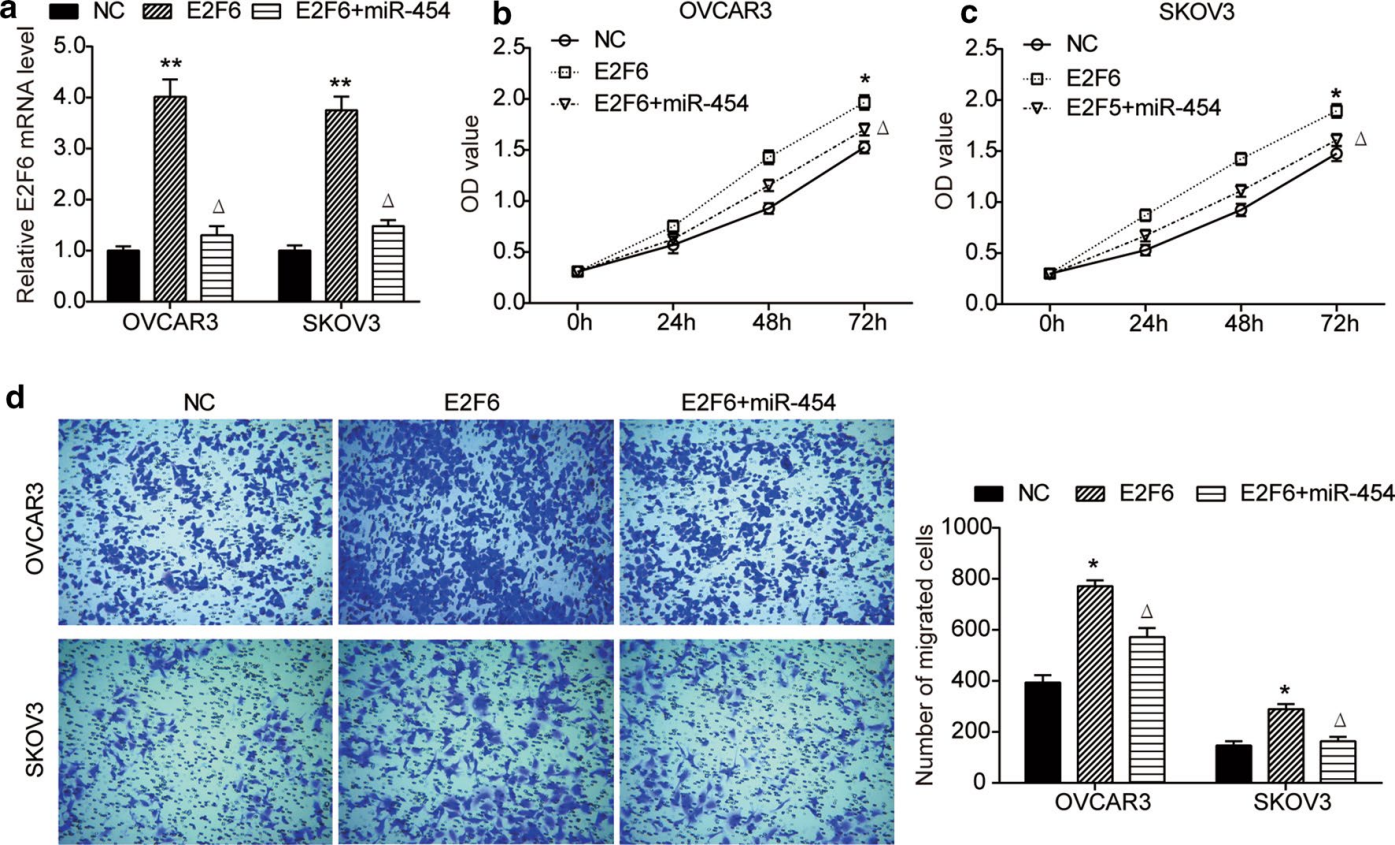
miR-454 overexpression abolishes the effects of E2F6 on the proliferation and migration of ovarian cancer cells. **a** Relative expression of E2F6 mRNA in OVCAR3 and SKOV3 cells co-transfected with pcDNA3.1-E2F6 and pCMV-MIR-miR-454 or transfected with pcDNA3.1-E2F6 alone was measured by RT-PCR, cells transfected with pcDNA3.1 vector was used as negative control (NC). **b**, **c** CCK8 assay was performed to assess the proliferation of OVCAR3 (**b**) and SKOV3 (**c**) cells after indicated transfection. **d** Migration ability of OVCAR3 and SKOV3 cells after indicated transfection. *P < 0.05, **P < 0.01 vs. NC group, ^Δ^*P *< 0.05 vs. E2F6 group

To investigate the role of E2F6 in miR-454-mediated progression of ovarian cancer cells, pcDNA3.1-E2F6 plasmid was constructed and transfected separately or co-transfected with pCMV-MIR-miR-454 into OVCAR3 and SKOV3 cells (Fig. 5a). As shown in Fig. 5b, c, E2F6 overexpression significantly enhanced the proliferation ability of OVCAR3 and SKOV3 cells compared with NC group, while miR-454 overexpression attenuated the promoting effect of E2F6 on cell proliferation. Moreover, the migration of OVCAR3 and SKOV3 cells was also promoted by E2F6 overexpression, which was also abolished by miR-454 overexpression (Fig. 5d). Collectively, E2F6 acts as a functional target of miR-454 in ovarian cancer.

## Discussion

Hypoxia is one of the important hallmarks of tumor microenvironment. Increasing studies have revealed that tumor hypoxia is closely related to the progression of cancer, such as tumor metastasis, recurrence and drug resistance [25–28]. It has been confirmed that hypoxia in vivo is associated with poor prognosis and high mortality in ovarian cancer patients, and involved in ovarian cancer cell proliferation [29, 30]. In the present study, we identified that miR-454 was markedly down-regulated by hypoxia in ovarian cancer cells, indicating that miR-454 may be associated with ovarian cancer. However, the functional role and underlying mechanism of miR-454 in ovarian cancer remain unclear.

Recently, emerging evidence has revealed that some miRNAs in serum have tumor relevance and tissue specificity as well as miRNAs in tissues, which could be also used as new targets and biomarkers for diagnosis, prognosis and treatment of cancers [31, 32]. miR-454 has been demonstrated to be dysregulated in cancers and involved in the growth and metastasis of several types of cancer. It has been revealed that miR-454 acts as an oncogene in triple negative breast cancer, and its high expression is associated with poor prognosis in patients with triple negative breast cancer [33, 34]. Fu et al. report that miR-454 is highly expressed in prostate cancer tissues and promotes prostate cancer cell proliferation and invasion by regulating NDRG2 [35]. However, in glioblastoma, Fang et al. demonstrate that miR-454 is down-regulated in tumors and cell lines, and miR-454 overexpression induces cell cycle arrest and inhibits cell proliferation [21]. miR-454 suppresses the growth, angiogenesis and metastasis of pancreatic ductal adenocarcinoma by targeting SDF-1 and LRP6 [36, 37]. These findings suggest that the expression of miR-454 is tissue-specific and may function as a biomarker for the diagnosis and prognosis of cancer. Herein, we found that the expression of miR-454 was significantly up-regulated in the serum of ovarian cancer patients compared with normal samples. Moreover, the expression of miR-454 was correlated with the clinicopathological stages of ovarian cancer. These results indicate that miR-454 could act as a potential diagnostic biomarker for ovarian cancer.

Applying gain-of-function experiments, our data revealed that miR-454 overexpression suppressed the proliferation, migration and invasion of ovarian cancer cells, and promoted cell apoptosis by regulating the Bcl-2/Bax axis and Caspase cascade. In addition, the Akt/mTOR and Wnt/β-catenin signaling pathways were both inhibited by miR-454 overexpression in ovarian cancer cells. Collectively, our data support the view that miR-454 functions as a tumor suppressor in ovarian cancer. Wang et al. recently show that miR-454 functions as a downstream target of lncRNA CCAT1, and miR-454 overexpression could promote cisplatin-induced apoptosis in A2780 cells [38], which is consistent with the promoting effect of miR-454 in apoptosis in this study. The controversial role of miR-454 might be due to different cancer cell types, sources and target genes [35]. Further study is required to confirm the precise role of miR-454 in the development of different types of cancer.

Numerous studies have indicated that miRNAs execute their function by regulating the expression of target genes [13, 39]. To determine the mechanism underlying the tumor-suppressive role of miR-454 in ovarian cancer, we identified the potential target gene of miR-454. As a member of E2F family of transcription factors, E2F6 plays critical roles in regulating cellular biological activities. Previous studies have revealed that E2F6 exerts an oncogenic role in the progression of cancers and functions as a target gene of some miRNAs [40–42]. Li et al. report that up-regulated expression of E2F6 is observed in gastric cancer tissues, and E2F6 knockdown inhibits cell proliferation and invasion [41]. In renal cell carcinoma, miR-425 suppresses cell proliferation and induces apoptosis by targeting E2F6 [40]. In the present study, by bioinformatics analysis and dual-luciferase reporter assay, we verified that E2F6 was a direct target of miR-454 in ovarian cancer cells, and miR-454 could negatively regulate the expression of E2F6. In addition, we demonstrated that E2F6 was significantly up-regulated in ovarian cancer tissues, and E2F6 overexpression significantly enhanced the proliferation and invasion abilities of ovarian cancer cells. Moreover, these changes triggered by E2F6 overexpression in ovarian cancer cells were abolished by miR-454 overexpression. Therefore, our findings suggest that miR-454 impedes the growth and metastasis of ovarian cancer by targeting E2F6.

## Conclusion

In summary, our data clearly for the first time demonstrated that miR-454 was up-regulated in serum of patients with ovarian cancer and correlated with clinicopathological stages, and miR-454 overexpression could inhibit ovarian cancer cell proliferation and invasion, and promote cell apoptosis, These data suggest that miR-454 acts as a tumor suppressor in the progression of ovarian cancer. Additionally, our study revealed that E2F6 was up-regulated in ovarian cancer and functions as a direct target of miR-454.The findings of the present study not only provide a new insight into the mechanism of ovarian cancer development but also suggest that the miR-454 may be a potential diagnostic biomarker and therapeutic target for ovarian cancer.

## Data Availability

The data supporting the conclusions of this paper are included within the manuscript.

## Abbreviations

Akt: Protein kinase B
CCK8: Cell counting kit-8 reagent
miRNA: MicroRNA
mTOR: Mammalian target of rapamycin
